# Controlling the Substrate Specificity of an Enzyme through Structural Flexibility by Varying the Salt-Bridge Density

**DOI:** 10.3390/molecules26185693

**Published:** 2021-09-20

**Authors:** Juan Huang, Qin Xu, Zhuo Liu, Nitin Jain, Madhusudan Tyagi, Dong-Qing Wei, Liang Hong

**Affiliations:** 1 State Key Laboratory of Microbial Metabolism, School of Life Sciences and Biotechnology, Shanghai Jiao Tong University, Shanghai 200240, China; juanhuang2015@sjtu.edu.cn; 2 Institute of Natural Sciences, Shanghai Jiao Tong University, Shanghai 200240, China; liuzhuo-chirality@hotmail.com; 3 School of Physics and Astronomy, Shanghai Jiao Tong University, Shanghai 200240, China; 4 Institute for Advanced Study, The Hong Kong University of Science and Technology, Hong Kong, China; 5 Department of Biochemistry and Cellular and Molecular Biology, University of Tennessee, Knoxville, TN 37996, USA; njain@utk.edu; 6 NIST Center for Neutron Research, National Institute of Standards and Technology (NIST), Gaithersburg, MD 20899, USA; madhusudan.tyagi@nist.gov; 7 Department of Materials Science and Engineering, University of Maryland, College Park, MD 20742, USA; 8 Peng Cheng Laboratory, Shenzhen 518055, China

**Keywords:** cytochrome P450, substrate specificity, structural flexibility, salt bridge

## Abstract

Many enzymes, particularly in one single family, with highly conserved structures and folds exhibit rather distinct substrate specificities. The underlying mechanism remains elusive, the resolution of which is of great importance for biochemistry, biophysics, and bioengineering. Here, we performed a neutron scattering experiment and molecular dynamics (MD) simulations on two structurally similar CYP450 proteins; CYP101 primarily catalyzes one type of ligands, then CYP2C9 can catalyze a large range of substrates. We demonstrated that it is the high density of salt bridges in CYP101 that reduces its structural flexibility, which controls the ligand access channel and the fluctuation of the catalytic pocket, thus restricting its selection on substrates. Moreover, we performed MD simulations on 146 different kinds of CYP450 proteins, spanning distinct biological categories including Fungi, Archaea, Bacteria, Protista, Animalia, and Plantae, and found the above mechanism generally valid. We demonstrated that, by fine changes of chemistry (salt-bridge density), the CYP450 superfamily can vary the structural flexibility of its member proteins among different biological categories, and thus differentiate their substrate specificities to meet the specific biological needs. As this mechanism is well-controllable and easy to be implemented, we expect it to be generally applicable in future enzymatic engineering to develop proteins of desired substrate specificities.

## 1. Introduction

Enzymes are often described to be highly specific in text books so that one enzyme can only catalyze one chemical reaction on a specific substrate [1]. However, in reality, many enzymes can catalyze multiple distinct types of chemical reactions on various substrates [1,2]. Of particular interest, even enzymes belonging to one family with rather similar structures can evolve to have drastically different substrate specificities so that some of them are primarily devoted on one single type of substrates, named here as “specialist”, while others can catalyze substrates of vastly different shapes and chemistry, denoted as “generalist” [3,4]. A typical example is cytochrome P450 (CYP450), a ubiquitous hemeprotein monooxygenase [3,5,6]. Thousands of crystal structures of CYP450s in both prokaryotes and eukaryotes are resolved experimentally and collected in the Protein Data Bank (PDB) [7,8], whose secondary structures and folding pattern are quite conservative, all containing 12 α-helices and four β-sheets or pairs of β-strands [9]. However, the substrate specificities of these CYP450s differ significantly so that most CYP450s in plants [10,11], bacterial species [12,13], and animals’ mitochondrion [14] exert a strong preference on a limited number of substrates, whereas those responsible for the metabolizing of drugs and pollutants in vertebrates can catalyze a range of chemically and structurally different substrates [15,16]. Understanding the underlying biophysical/biochemical mechanism that evolves the structurally similar enzymes to possess different substrate specificities is of great importance for structural biology and enzymatic engineering.

As reported in the literature, many different mechanisms have been adopted by enzymes to tune their substrate specificities [1], e.g., the catalytic pocket assuming different conformations [17], blocking part of the pocket by small dummy molecules [12], hydrogen-bonding of the substrates to different residues and solvent molecules in the pocket [18], as well as mutagenesis [19], etc. A consensus has been reached that greater conformational diversity or flexibility can broaden the substrate specificities of an enzyme [1,17] so that the residues forming the catalytic pocket could take different conformations to fit, bind, and catalyze the distinct substrates. However, the microscopic mechanisms reported were often system specific, which is heavily dependent on the unique local structure in the enzymes. The intriguing question arises as to whether there exists a robust method that can be generally applied to tune the substrate specificities of proteins or even partition the enzymes in one superfamily with highly conserved structures to possess drastically different substrate specificities, i.e., generalist versus specialist.

**Figure 1:**
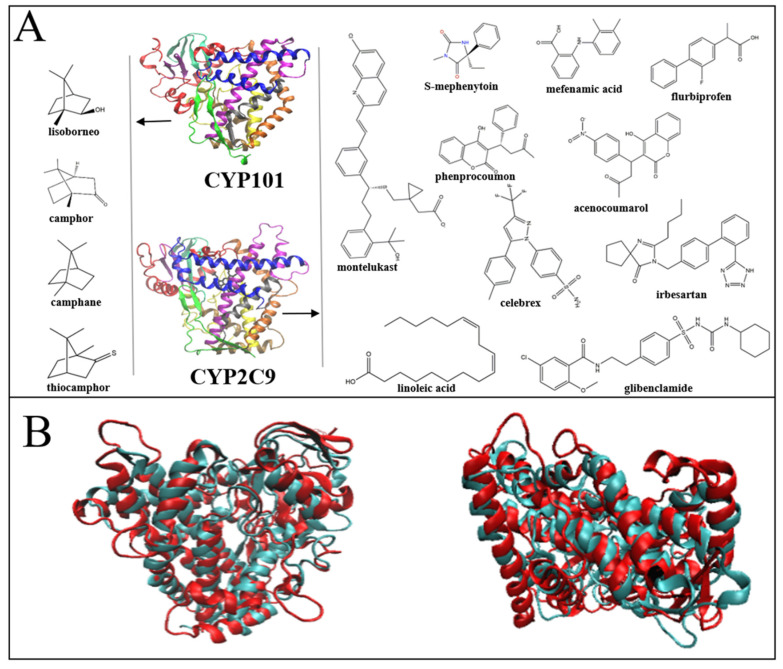
Comparison of the structures and ligands of CYP101 and CYP2C9. (**A**) Comparison of substrate specificities between CYP101 and CYP2C9. The substrates of CYP2C9 contain many different small molecules, long-chain polyunsaturated fatty acids and high molecular weight aromatic compounds, while CYP101 only catalyzes a limited number of small compounds, mostly camphor and camphor derivatives. (**B**) Superposition of the backbones of bacterial CYP101 (cyan, PDB 1DZ9) with that of the human CYP2C9 (red, PDB 5XXI) from the top view (left) and the side view (right).

In the present work, we focus on a comparison between a typical bacterial CYP450 enzyme, CYP101, and a representative human CYP450 enzyme, CYP2C9. The bacterial CYP101 from *Pseudomonas putida* has a strong selectivity on the substrates, mostly camphor or a few camphor derivatives [20], and it is one of the most studied CYP450, serving as a prototype for CYP450 structure-function studies [21,22]. In contrast, CYP2C9, often found in human liver, metabolizes a wide variety of xenobiotics and endogenous compounds [23] (Figure 1A). In spite of their highly distinct substrate specificities, the secondary and tertiary structures of these two CYP450s are rather similar (Figure 1B). By combining neutron scattering and molecular dynamics (MD) simulation, we found that, as compared to CYP101, CYP2C9 has a much larger degree of structural flexibility both globally and locally, especially around the gating area of the substrate access channel and the catalytic pocket, crucial for accommodating substrates of distinct sizes and shapes. By further examining the protein structures, we identified the density of salt bridge as the primary cause for the control of the structural flexibility of the two CYP450s; a greater amount of salt bridges in CYP101 renders stronger constraints on the internal motion, particularly at the F-G region, which works as a lid of the catalytic pocket. Moreover, we also applied MD simulations on all 146 kinds of CYP450 enzymes, whose crystal structures are publicly available, and found the above mechanism is generally valid in this superfamily; the density of salt bridge controls protein flexibility and, thus, its substrate specificity.

## 2. Results and Discussion

### 2.1. Different Structural Flexibilities between CYP101 and CYP2C9

**Figure 2:**
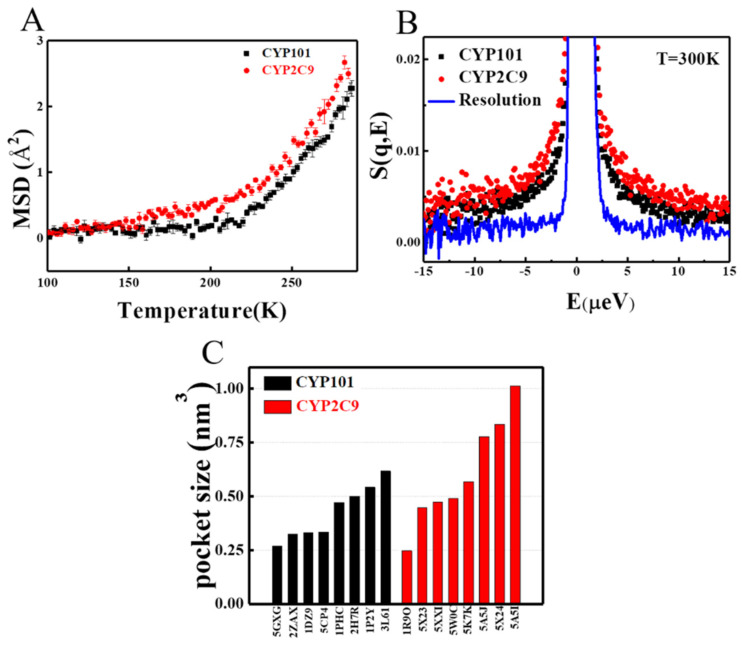
Comparison of the flexibilities between CYP101 (black) and CYP2C9 (red). (**A**) Mean-squared atomic displacement (MSD) as a function of temperature from 4 K to 290 K measured by neutron scattering. Error bars represent one standard deviation. (**B**) Quasi-elastic neutron spectra measured at 300 K, in which the blue line is the resolution function collected on vanadium. More detailed information on the experimental setup and sample preparation can be found in Materials and Methods. (**C**) The variations of pocket volume in crystal structures of CYP101 and CYP2C9, when binding to different substrates (see details in Figure S1). Here, the volume is calculated using the Pocket Volume Measurer (POVME) program. Examples to illustrate the pocket volume determined by the software are presented in Figure S2.

As seen in Figure 1A, CYP2C9 can catalyze a much broader range of chemically and structurally different substrates as compared to CYP101, implying that CYP2C9 should have much greater structural flexibility. To examine this, we performed neutron scattering on these two CYP450s hydrated in D_2_O. As neutron is highly sensitive to hydrogen atoms, the measured signals, thus, primarily reflect the dynamics of proteins [24,25,26]. As evident by Figure 2A, CYP2C9 has significantly larger mean-squared atomic displacement (MSD), i.e., much more flexible, as compared to CYP101 above 150 K. We note that, at lower temperatures, proteins will behave as harmonic solids without functions [27,28]. In addition to MSD, we also measured the quasi-elastic neutron spectra over the energy range from 1 μeV to 15 μeV, denoted as S(q, E), which furnishes the distribution of the dynamical modes in the time window from 60 ps to 1 ns. As can be seen in Figure 2B, the S(q, E) of CYP2C9 is significantly broader than that of CYP101, revealing that the internal motion of the former is faster. Moreover, we examined the local flexibility of the two CYP450s at the catalytic pockets by comparing their crystal structures when binding to different substrates. As can be seen in Figure 2C, the pocket volume of CYP2C9, calculated using the Pocket Volume Measurer (POVME) program [29,30], can change from 250 to 1000 Å^3^, while that of CYP101 is limited in the range from 250 to ~600 Å^3^. Hence, the catalytic pocket of CYP2C9 has much greater flexibility to accommodate distinct substrates.

**Figure 3:**
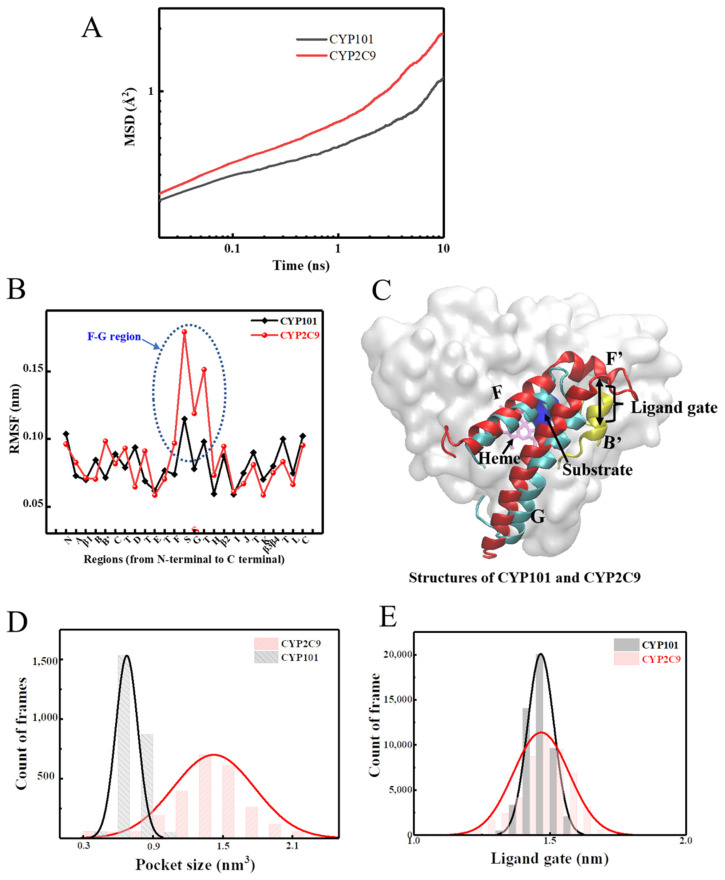
MD-derived flexibilities in CYP101 and CYP2C9 obtained by analyzing three independent 500 ns simulations for each protein. (**A**) Global flexibilities indicated by mean-squared atomic displacement (MSD) (10 ps to 10 ns). (**B**) Local flexibilities suggested by root-mean-square fluctuation (RMSF) clustered on the secondary structures, where the blue dotted circle highlights the most evident difference, appearing at the F–G region. We note that the symbol “S” marked in red corresponds to a linker connecting F and G helices. This linker is composed by coils and F’ helix in CYP2C9 but only a short coil in CYP101 (see (**C**)). (**C**) Superposition of the crystal structures of CYP101 (cyan, PDB 1DZ9) and CYP2C9 (red, PDB 5XXI) with the F–G region and B’ helix (yellow) highlighted. The ligand gate controlling the opening and closing of the ligand access channel is formed by the B’ helix and the linker connecting F and G helices, and it is marked by a black double-ended arrow. The positions of heme (purple) and the substrate (blue) are also marked out. The distribution of the fluctuations of (**D**) the volume of the catalytic pocket and (**E**) the distance of the ligand gate observed in MD.

To complement the experimental studies, three independent 500 ns long all-atom molecular dynamics (MD) simulations on each of the two proteins, CYP101 and CYP2C9, were conducted. Detailed simulation protocols are provided in Materials and Methods. The results of mean-squared atomic displacement (MSD) for each trajectory are presented in Figure S5 showing that results obtained from each trajectory are as similar as each other for a given protein. To improve statistics, the analysis for both CYP101 and CYP2C9 was conducted by averaging over the three 500 ns trajectories. Indeed, CYP2C9 has greater overall mobility as evidenced by the larger MSD derived from MD simulations in Figure 3A, confirming the observations of the neutron experiments (Figure 2A). To identify the local regions that mostly contribute to the difference in the structural flexibilities between the two proteins, we compared the root-mean-square fluctuation (RMSF) of their local structures. The comparison presented in Figure 3B is based on the secondary structures instead of residues, as the two CYP450s share similar secondary structures while baring low sequence identity (19.22%). As can be seen, the major dynamical difference of the two CYP450s lies in the F–G region, which is composed of the long helices of F and G, and the linker between them. This F–G region serves as the lid to cover the catalytic pocket, and the linker connecting F and G helices together with the B’ helix gates at the entry of the ligand into the catalytic pocket [31,32,33,34] (see Figure 3C). In CYP101, the linker is a short peptide chain (F–G loop), while it is a long coil plus a small helix F’ in CYP2C9 that can adopt much more conformational changes (Figure 3C). As a result, the much greater flexibility of the whole F–G region in CYP2C9 renders larger fluctuation at the catalytic pocket (Figure 3D and Figure S2 in Supplementary Materials, SM) and at the gate of the ligand access channel (Figure 3E) to accommodate different substrates.

In summary, the analyses of the dynamical neutron experimental data, crystal structures, and MD simulations of the two proteins reveal that CYP2C9 has higher structural flexibility than CYP101, both globally and locally, especially around the ligand gate and the catalytic pocket. The higher structural flexibility of CYP2C9 allows the width of the ligand gate and the volume of the catalytic pocket to assume larger variations to accept many distinct substrates. In contrast, CYP101 has lower structural flexibility, which may limit its option on substrates (camphor or camphor derivatives). However, this could also lead to a better conformational match between the lock and the key [35] (i.e., the ligand and the enzyme pocket), rendering the specialist (e.g., CYP101), higher enzymatic efficiency on its devoted substrate, as compared to the generalist (e.g., CYP2C9) [36,37,38].

### 2.2. The Role of Salt Bridges in Controlling the Flexibility of CYP101 and CYP2C9

**Figure 4:**
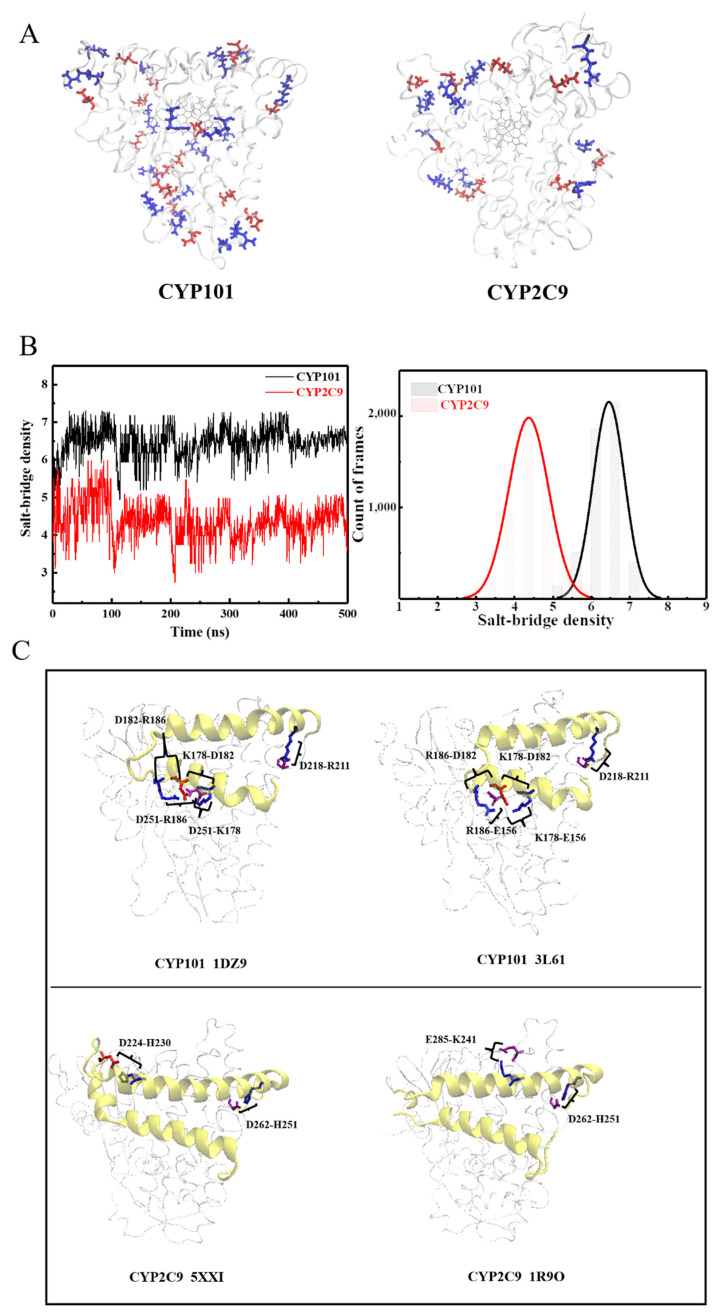
Comparison of salt bridges in CYP101 and CYP2C9. (**A**) The salt bridges with life time more than 20% of the simulation time in the protein structures of CYP101 (PDB: 1DZ9) and CYP2C9 (PDB: 5XXI), with the negatively and positively charged residues colored in red and blue, respectively. (**B**) The fluctuation of the salt-bridge densities over time observed in MD for CYP101 and CYP2C9, and the resulting distribution. (**C**) The salt bridges in the F–G region (yellow) of the crystal structures of CYP101 (PDB: 1DZ9 and 3L61, top) and those in CYP2C9 (PDB: 5XXI and 1R9O, bottom). We note that further analysis of more crystal structures confirmed that the conclusion that CYP101 has more salt bridges around the F–G region is generally valid. The results are not shown here.

The intriguing question thus arises as to why CYP2C9 has such great structural flexibility, especially around the ligand gate and the pocket area. As generally recognized, the protein internal mobility is often controlled by inter-residue interactions, such as intra-protein hydrogen bonds [39], salt bridges [40,41,42], and hydrophobic interactions [43,44] (mostly interactions between aromatic residues), as well as protein-water hydrogen bonds [45,46,47]. By thoroughly comparing the structures of the two CYP450s in Figure S3 of SM, we can unambiguously exclude the factors: hydrophobic interactions (Figure S3A,B and Table S1), intra-protein hydrogen bonds (Figure S3C), and protein–water hydrogen bonds (Figure S3D) as the cause for the dynamical difference observed between the two CYP450s. Subsequently, we analyzed the salt bridges in the two proteins. As can be seen in Figure 4A and Table 1, CYP101 has many more salt bridge pairs. The salt-bridge density, defined as the number of salt bridges per 100 amino acids in a protein, is 7.4 as found in 500 ns simulations of CYP101 (PDB: 1DZ9), more than twice of that (3.5) in CYP2C9 (PDB: 5XXI). Such a drastic difference in salt-bridge density is also confirmed in MD simulations, by which it is found to be 5–8 in CYP101, much higher than the value of 3–6 in CYP2C9 (Figure 4B). As a salt bridge furnishes strong inter-residue interactions (~3–5 kcal/mol) [48,49,50], its greater population in CYP101 will inevitably lower the global flexibility of the protein (see Figure 2 and Figure 3A).

Such a difference in salt-bridge density is also evident locally at the F–G region. As can be seen in Figure 4C, five salt bridges appear at the F–G region in CYP101, while only two could be found at the corresponding region of CYP2C9. Furthermore, the positively charged residues constituting the two salt bridges in CYP2C9 are mainly histidine, which normally form weaker salt bridges as compared with the residues lysine and arginine found in CYP101. Moreover, a salt-bridge network is formed in CYP101 both within the F-helix (residues K178 and R186 to D182) and between the F-helix and the enzymatic core (residues K178 and R186 in F-helix to D251 in PDB 1DZ9 or to E156 in PDB 3L61 in the core) (see Figure 4C). This network can firmly anchor the F-helix onto the enzyme core. Therefore, all of the above characteristic features of the salt bridges in the F–G region of CYP101: greater density, stronger interacting strength, and the specific networking structure, will drastically limit the movement of the F–G region in CYP101, causing its stronger selection on the size and shape of the substrates.

Furthermore, we calculated the life time of each salt bridge in both CYP101 and CYP2C9 (see Figure S4A,B and Table 1, respectively). In Figure S4C, we further divided salt bridges into three categories: short, medium, and long-lived. They correspond to the salt bridge being formed for less than 2% (shorter than 10 ns), 2% to 20% (10 ns to 100 ns), or more than 20% (longer than 100 ns) of the entire simulation time (500 ns), respectively. As can be seen, CYP101 has significantly more salt bridges than CYP2C9 in all three categories. In Figure S4D,E, we have highlighted the long-lived salt bridges in these two proteins. As can be seen, CYP101 have more long-lived salt bridges both globally (marked as bonds) and locally in the F–G region (marked as spheres), which control the ligand access and fluctuation of the enzymatic pocket. In particular, two pairs of long-lived salt bridges, ASP218-ARG211 with a life time of 43.46% (217.3 ns) and ASP251-LYS178 with a life time of 49.74% (248.7 ns), are identified in the F–G region of CYP101, while only one is found in the F–G region of CYP2C9, ASP262-HIS251, with a life time of 41.10% (205.5 ns). Both ASP218-ARG211 in CYP101 and ASP262-HIS251 in CYP2C9 are located at the same sites in the protein structures and contribute to the stabilization of G-helix, whereas ASP251-LYS178 in CYP101 is formed via the connection of LYS178 in the F-helix and ASP251 in the I-helix, which can strongly rigidify the F–G region by anchoring the F-helix on the I-helix. Thus, one could deduce that this particular salt bridge (ASP251-LYS178) might play an important role in reducing the flexibility of the motion of the functional F–G region in CYP101 with respect to CYP2C9.

### 2.3. The Generality on the Mechanism That Salt-Bridge Density Determines the Protein Flexibility and, Thus, Its Substrate Specificity across the CYP450 Family

**Figure 5:**
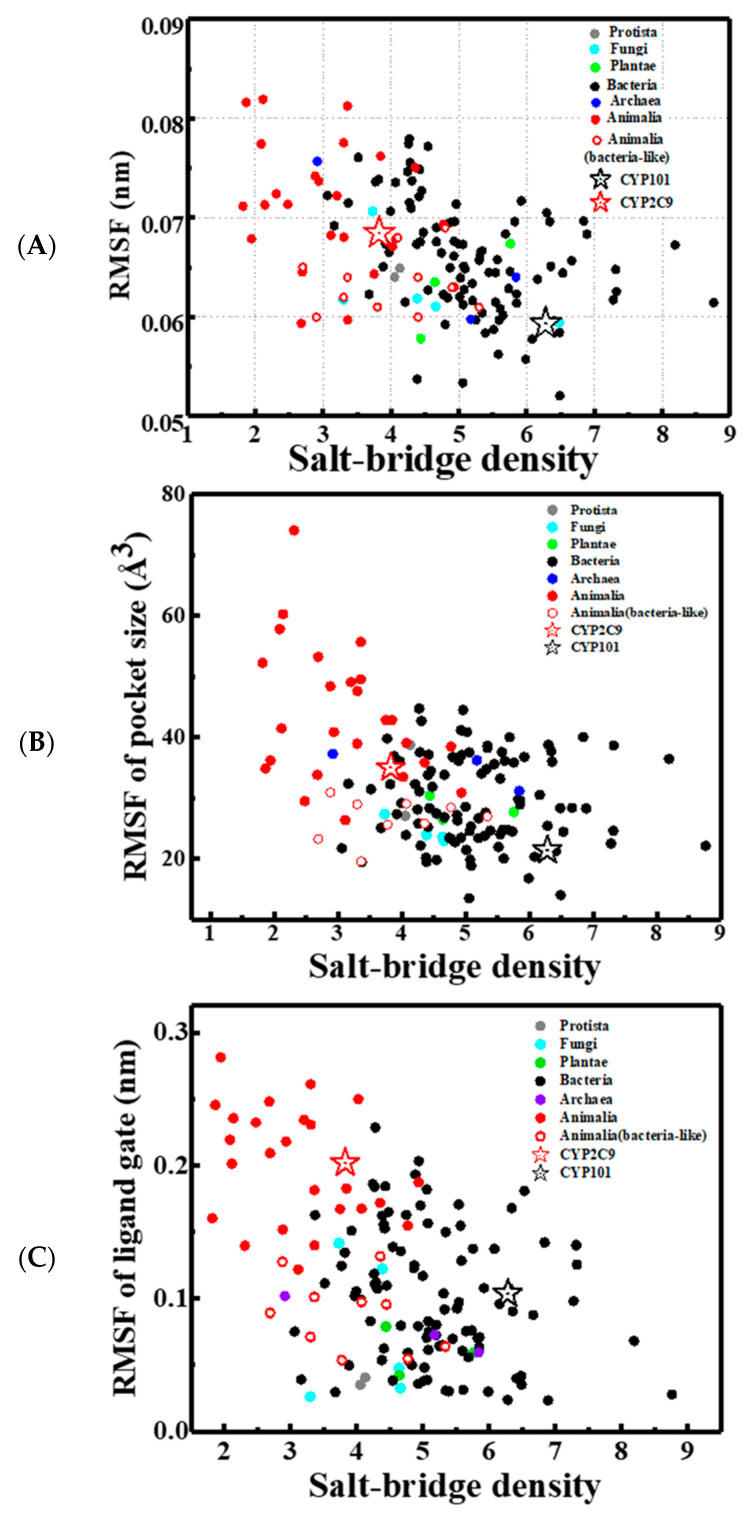
The correlation between the salt-bridge densities, i.e., the number of salt bridges per 100 residues in the protein, with (**A**) the overall internal mobility of the enzyme, defined as the room-mean-squared fluctuation (RMSF), (**B**) the flexibility of the catalytic pocket, defined as the room-mean-squared fluctuation of pocket volume, and (**C**) the flexibility of ligand gating area, defined as the room-mean-squared fluctuation of the distance of the ligand gate from F/G loop to the B’ helix (e.g., the CYP101 was defined by the distance between Ser190 in the F/G loop and Pro89 in the B’ helix). Here, 146 different kinds of CYP450 proteins were studied, and 10 ns MD trajectories were conducted for each protein to obtain these quantities. The data are colored by their biological categories: Fungi (cyan), Archaea (blue), Bacteria (black), Protista (grey), Animalia (red solid spheres), and Plantae (green), with CYP101 and CYP2C9 highlighted by starts and several bacteria-like animal CYP450s labeled by red empty circles. The Pearson Correlation Coefficient [52] for (**A**–**C**) are −0.43, −0.38, and −0.46, respectively, which are statistically meaningful and qualitatively supports the hypothesis that the salt bridges play an important role in restraining the structural flexibilities for all CYP450 proteins in the superfamily.

A further question arises as to whether the mechanism discovered above that salt-bridge density determines the structural flexibility and, thus, the substrate specificity also applies to other CYP450s. We have searched through the PDB data bank, which contains thousands of CYP450 structures. We classified these structures into 146 types based on the CYP450 Nomenclature given in Reference [51]. For each type of CYP450, a representative crystal structure with the most complete PDB structure was chosen for study by a 20 ns MD simulation (see simulation details in Materials and Methods and Table S2). We analyzed the correlation of salt-bridge density with the overall protein internal mobility, and also with the flexibility of the catalytic pocket and of the ligand gating area among different P450s using MD simulations. The results are presented in Figure 5A–C, respectively. We found that higher density of salt bridges will not only reduce the overall enzyme mobility but also decrease the flexibility of the ligand gating area and catalytic pocket.

Given that CYP450s belonging to different biological categories show distinct substrate specificities [3], it is highly desirable to compare the flexibilities and salt-bridge densities of the CYP450s among biological categories. As shown in Figure 5, distinct biological categories are colored differently: Fungi (cyan), Archaea (blue), Bacteria (black), Protista (grey), Animalia (red), and Plantae (green). As the data for Fungi, Archaea, Protista, and Plantae are too limited, we here focus on the comparison between bacterial (black) and animal CYP450s (red, mainly from vertebrates). As seen in Figure 5, bacterial CYP450s have more salt bridges and less mobility, i.e., smaller RMSF, as compared to the animal ones. We can observe that the salt-bridge density shows a similar impact on the flexibility of the ligand gate distance and the catalytic pocket size with the averaged RMSF. Meanwhile, these two categories of CYP450s also have quite different spectra of substrates [20,23,53]. Most CYP450s in bacteria have been evolved in a highly specialized environment and optimized for the biosynthesis of specific metabolites. A hypothesis here is that the evolution of the bacterial CYP450 might make use of a high density of salt bridges to lower their structural flexibility so as to fit better to their specific substrate and, thus, improve the corresponding catalytic efficiency. On the other hand, similar to CYP2C9, most of the animal CYP450s are found in more advanced organs such as the human liver, and mainly work as a “generalist” to catalyze the detoxification of distinct types of alien molecules [15,16]; they require significant structural flexibility on the substrate-heme pocket to accommodate various metabolites binding in different poses. These typical generalists (solid red spheres in Figure 5) tend to have lower salt-bridge densities and higher RMSF as found in MD. It is of particular interest to note that the substrate specificities of a few CYP450s in Animalia deviate from those of most animal ones. They primarily catalyze a limited number of substrates in the same way as bacterial CYP450. These bacteria-like animal CYP450s are highlighted by empty red circles in Figure 5, e.g., CYP11A and CYP11B in the steroid biosynthetic pathways, as well as CYP24 and CYP27A in the cholesterol–bile acid biosynthetic pathway, which are found exclusively in the inner mitochondrial membrane [15]. The presence of these bacteria-like animal CYP450s was explained as a gene transfer [54] from ancient prokaryotes into high level eukaryotes, or possibly a convergent evolution [55]. As can be seen in Figure 5, the bacteria-like animal CYP450s (empty red circles) possess a greater amount of salt bridges and lower structural flexibility than other animal ones (solid red spheres), in agreement with the mechanism we proposed. In summary, the comparison of flexibility, salt-bridge density, and substrate specificity among bacterial (black spheres), normal animal (solid red spheres), and bacteria-like animal CYP450s (empty red circles) suggests that salt-bridge density plays an important role in that it regulates the internal flexibility of CYP450 and, thus, its substrate specificity in the superfamily. 

As revealed in the present work, the CYP450 superfamily uses salt-bridge density to vary the structural flexibility of proteins in different biological categories and, thus, differentiate their substrate specificities to meet the specific biological needs. This could be a good example to illustrate how enzymes adapt to different environmental needs by changing their structural flexibilities through a generalized and well-controlled chemical method. This could also be informative for enzymatic engineering. For example, critical enzymes of microbes for biofuel production and plastic degradation might need to tolerate some diversity in their substrates [56], while the key enzymes for biosynthesis would like to exclude promiscuity and only produce one product as pure as possible. The present study provides a relatively simple guideline for enzymatic engineering, e.g., reducing salt bridges for the former to increase its flexibility to accommodate different substrates while adding salt bridges for the latter to enhance its specificity and, probably, the enzymatic efficiency.

## 3. Materials and Methods

### 3.1. Protein Expression and Purification

In this study, the protein CYP101 was expressed and purified following the methods described in Reference [57].

In order to improve the solubility and expression of CYP2C9, the N-terminal transmembrane domain of CYP2C9 (residues 1–29) was replaced by a highly charged short polypeptide MAKKTSSKGR, and the segments LPVIGNILQI and GIFPLA were replaced by PLVGSLPFLP and PQMATL, respectively. In addition, a four-histidine tag was introduced at the C-terminus to facilitate protein purification.

The gene sequence of the revised CYP2C9 was synthesized and subcloned into a pCWori vector, which was transformed into *Escherichia coli* XL1 blue cells. A single colony of these transformed cells was selected from an LB/Agar plate containing 100 μg/mL of Ampicillin incubated overnight at 37 °C, and then incubated in a 50 mL of LB medium containing 100 μg/mL of Ampicillin at 37 °C and shaken at 250 rpm overnight. Then 8 mL of the incubated LB medium was seeded into 4 L of Terrific broth medium to be incubated at 37 °C and shaken at 220 rpm for 3~4 h until the culture reached an OD_600_ of roughly 0.8. After adding δ-aminolevulinic acid and isopropyl-β-D-thiogalactoside into the solution till they reach the concentrations of 1.0 mM and 0.5 mM, respectively, the medium was further incubated at 30 °C and shaken at 220 rpm for 48 h. The cells were harvested by centrifugation and resuspension in 300 mL buffer A (20 mM KPi, 20% glycerol, 1 mM phenylmethanesulfonyl fluoride, and 10 mM β-mercaptoethanol). The resulting solution was then subjected to sonication followed by high-speed centrifugation (18,000 rpm) for 30 min. The supernatant was loaded onto a Ni-NTA resin (Qiagen, Valencia, CA, USA) that was pre-equilibrated with 150 mL of buffer B (500 mM KPi, pH 7.4, and 20% glycerol) to allow binding of CYP2C9. The resin was washed by 40 mL of buffer C (500 mM KPi, pH 7.4, 20% glycerol, 0.5 mM phenylmethanesulfonyl fluoride, and 10 mM β-mercaptoethanol), then by 40 mL of buffer D (100 mM KPi, pH 7.4, containing 100 mM NaCl, 20% glycerol, 0.5 mM phenylmethanesulfonyl fluoride, and 10 mM β-mercaptoethanol), and thirdly by 50 mL of buffer E (10 mM KPi, pH 7.4, 3100 mM NaCl, 20% glycerol, 0.5 mM phenylmethanesulfonyl fluoride, 10 mM β-mercaptoethanol, and 1 mM histidine). Finally, the protein was eluted by buffer F (10 mM KPi, pH 7.4, 100 mM NaCl, 20% glycerol, 10 mM β-mercaptoethanol, 1 mM phenylmethanesulfonyl fluoride, and 30 mM histidine). Peak fractions were pooled and diluted using 150 mL of buffer G (5 mM KPi, pH 7.4, 20% glycerol, 1 mM EDTA, 1 mM phenylmethanesulfonyl fluoride, and 0.2 mM DTT). The diluted solution was then loaded onto 5 mL of CM-resin that had been equilibrated with the same buffer G, and then washed by 75 mL of buffer G. In the end, the CYP450 proteins were eluted by buffer H (50 mM KPi, 500 mM NaCl, 20% glycerol, 1 mM EDTA, and 0.2 mM DTT, pH 7.4). To obtain CYP450 of sufficiently high purity, one needs to make sure that the ratio of the fluorescence intensities between two wavelengths (417/280)_nm_ measured by UV-visible spectroscopy is greater than 1.4.

### 3.2. Neutron Scattering Experiment

The purified protein powder for the neutron scattering experiment was prepared by dialysis and lyophilization, where the protein was extensively dialyzed against H_2_O at least 4 times to remove buffer salts. Deuterium exchange was initiated by dissolving dry powder in D_2_O and incubated at 4 °C for 2 h to ensure any exchanged hydrogen was fully exchanged by deuterium, and all samples needed to be lyophilized to keep the protein in a dry form. Then, the water adsorption process was operated in a glove box with inert gas purged to avoid the disturbance of water in the air. The ultrapure water (H_2_O) was supplied by a Millipore Direct-Q system (18.2 MΩ·cm at 25 °C), and the heavy water (D_2_O) was purchased from Sigma-Aldrich. The protein sample was sealed in a desiccator with D_2_O to adsorb water in the glove box. The hydration levels of protein were estimated by measuring the sample weights before and after water adsorption. The final hydration level of the samples was about 0.4 g water/gram protein. The overall weight was about 140 mg for each sample.

Both elastic and quasi-elastic incoherent neutron scattering spectra were collected on ligand-free CYP101 and CYP2C9 samples at *h* = 0.4 (0.4 gram water per gram protein) using the NG2 high-flux backscattering spectrometer at NIST Center for Neutron Research at National Institute of Standard and Technology with a fixed energy resolution of ~0.8 μeV (corresponding to a time resolution of ~1 ns) [27,58]. Elastic scans were performed for both protein samples in the temperature range of 4~290 K at a heating rate of 1.0 K/min. No correction for multiple scattering was needed since the neutron transmission was over 0.9, and multiple scattering was, thus, negligible [59]. The experimentally measured quantity in the elastic neutron scattering is the so-called elastic intensity, i.e., the intensity of the peak in the dynamic structure factor, S(q, Δt), as a function of temperature, where Δt is the instrument resolution, which is 1 ns here. The average mean-squared atomic displacement (MSD) (Figure 2A) is obtained by applying a *q*^4^ fitting algorithm on S(q, Δt) in the *q* range of 0.25–1.75 Å^−1^. This fitting algorithm was proposed in Reference [60]. In addition to elastic scan, we also measured the quasi-elastic neutron scattering spectra using the same instrument. They were conducted at 300 K for both proteins, and the spectra presented in Figure 2B averaged the *q* range from 0.5 to 1.7 Å^−1^.

### 3.3. Molecular Dynamics (MD) Simulation

The simulations were performed by GROMACS version 5.1.2 [61] with the periodic boundary condition. We used the force field CHARMM36 [62] for the protein and the TIP3P [63] model for water. All bonds involving hydrogen atoms were constrained with LINCS algorithm [64] to allow a time step of 2 fs. The Particle Mesh Ewald (PME) method [65] was applied for the electrostatic interactions with a real space cutoff of 12 Å, while the Van der Waals interactions were switched to zero gradually from 10 Å and truncated at 12 Å. The systems were energy minimized with the steepest descent method to a convergence of maximal force of 10 kJ·mol^−1^nm^−1^ or maximum 50,000 steps. Then the system was equilibrated, firstly with heavy atoms restrained using a force constant of 1000 kJ·mol^−1^nm^−2^ while hydrogen atoms, the solvent, and ions were allowed to evolve under the NVT condition for 10 ns at 300 K, followed by a 10 ns NPT simulation at 300 K and 1 atm with all atoms released. The temperature coupling is realized using the velocity rescaling scheme [66] with the coupling time constants τ = 0.1 ps and the pressure coupling is performed using Parrinello–Rahman scheme [67] with τ = 0.4 ps. Under the same NPT condition, the equilibrated systems were applied to three independent 500 ns MD simulations for production on the model of CYP101 based on the crystal structure 1DZ9 and that of CYP2C9 based on 5XXI, whose analyses are presented in Figure 3 and Figure 4, Figures S3–S5, and Table 1.

Similar MD simulation setups at 300 K were applied on all other CYP450 family members. All cytochrome CYP450 structures were collected from the RCSB protein data bank (PDB) (https://www.rcsb.org/ 10 May 2019). Up to April 2019, all crystal structures were classified into 146 kinds of CYP450s based on CYP450 Nomenclature given in Reference [57]. For the analysis in Figure 5, a representative crystal structure in each kind of CYP450 with most complete PDB structure was chosen and studied by a 20 ns MD simulation. In order to compare the flexibilities of the CYP450s under the same conditions, any ligands in these structures was removed for the MD simulations. As we found that the structures of the CYP450s have been equilibrated after the first 10 ns of the simulations, the second 10 ns of the trajectories were used for further analysis and the results are presented in Figure 5. The value of the salt-bridge density, defined as number of salt bridges per 100 residues in the protein, is averaged over the last 10 ns trajectories of the MD simulation performed on each CYP450. The RMSF is obtained by analyzing the same portion of the trajectory, and it is averaged over all residues in each protein.

A salt bridge is considered to be formed if the distance between any of the oxygen atoms of acidic residues and the nitrogen atoms of basic residues are within the cutoff distance (default 3.2 Å) in at least one frame. The life time s is defined as the portion of the trajectory, during which the salt bridge is formed, following the method from Karshikoff and Jelesarov [68]. Other structural properties such as root-mean-square fluctuation (RMSF), salt-bridge pairs, and aromatic residues clusters, as well as the distance between B’ helix and the linker between F and G helices were calculated using GROMACS standard analysis tools and VMD [69]. The pocket volume of the active sites were calculated using POVME 2.0 [29,30].

## 4. Conclusions

The CYP450 superfamily contributes a broad array of biological functions in living organisms. Despite dramatic differences in the substrate specificity among CYP450 enzymes, all of them share similar protein fold and constitution of secondary structures. Herein, by performing a neutron scattering experiment and molecular dynamics simulation on CYP2C9 and CYP101, we showed that the higher population of salt bridges suppresses the global and local flexibility of CYP450 and, thus, limits its option on substrates. Furthermore, we examined the relationship between the salt-bridge density and the flexibility of all 146 types of CYP450 enzymes, whose crystal structures are available in the PDB databank, using MD simulations, and found that the negative correlation between them is generally valid for the entire superfamily. More importantly, we identified that such a difference in structural flexibility is important to divide these CYP450s into substrate generalists and specialists. Thus, the present work proposed a general mechanism that a superfamily of enzymes can make use of a simple chemical method by controlling the density of salt bridges to evolve enzymes of similar structures towards drastically different substrate specificities to fit the biological need. These findings could be of great impact for the future design of specific CYP450s or other enzymes to meet different industrial demands. As this mechanism is well-controllable and easy to be implemented, we expect it to be generally applicable in future enzymatic engineering to develop proteins of desired substrate specificities.

## Figures and Tables

**Table 1:** The average life time of salt-bridge pairs in CYP101 and CYP2C9 obtained from 500 ns simulations.

CYP450s	CYP101 (PDB 1DZ9)	Life Time (%)	CYP2C9 (PBD 5XXI)	Life Time (%)
Salt bridge	ASP89-LYS84	0.40	ASP89-LYS84	3.06
pairs	GLU155-LYS158	0.78	GLU155-LYS158	3.86
	ASP341-ARG335	1.04	ASP341-ARG335	13.84
	ASP224-HIS230	1.74	ASP224-HIS230	14.66
	GLU325-ARG329	2.02	GLU325-ARG329	14.70
	GLU274-LYS270	2.72	GLU274-LYS270	16.06
	GLU122-ARG125	6.76	GLU122-ARG125	18.72
	ASP143-ARG139	8.52	ASP143-ARG139	26.40
	GLU354-ARG357	13.40	GLU354-ARG357	27.86
	ASP262-HIS251	14.38	ASP262-HIS251	41.10
	GLU147-ARG139	15.68	GLU147-ARG139	45.02
	GLU405-HIS396	17.40	GLU405-HIS396	49.74
	GLU354-HIS411	19.50	GLU354-HIS411	52.62
	GLU85-ARG377	22.12	GLU85-ARG377	65.72
	GLU92-LYS121	24.62	GLU92-LYS121	98.64
	GLU148-ARG186	31.38	GLU148-ARG186	98.66
	ASP316-HIS17	32.78		
	GLU237-ARG231	36.02		
	GLU287-ARG342	38.04		
	ASP25-ARG57	39.34		
	ASP218-ARG211	43.46		
	GLU286-ARG364	48.28		
	ASP251-LYS178	49.74		
	GLU273-ARG280	52.28		
	GLU262-LYS266	58.90		
	GLU331-ARG72	60.94		
	GLU140-ARG143	69.22		
	ASP236-ARG240	77.78		
	ASP380-ARG271	87.16		
	ASP77-HIS80	95.50		
sum	30		16	
Salt-bridge density	7.4		3.5	

## Data Availability

The data presented in this study are available on request from the corresponding author.

## Supplementary Materials

Detailed information on ligands of selected crystal structures of CYP101 and CYP2C9, comparison of the catalytic pocket between CYP101 and CYP2C9 and comparison of the contributions from other structural factors to the flexibility of CYP101 and CYP2C9, the life time of salt-bridge pairs in CYP101 and CYP2C9, MSD (10 ps to 10 ns) in CYP101 and CYP2C9 obtained by analyzing three independent 500 ns simulations for each protein, aromatic residues in CYP101 and CYP2C9, available experimental structures of CYP450s that were investigated by MD simulations, and a list of the bacteria-like animal CYP450s in Figure 5. References [70,71] are cited in the supplementary materials.

## Sample Availability

Samples of the compounds are available from the authors.
