# Supplementary material for: Controlling the Substrate Specificity of an Enzyme through Structural Flexibility by Varying the Salt-Bridge Density
Source: Molecules. 2021 Sep 20;26(18):5693. doi: 10.3390/molecules26185693 (PMC8470667; doi:10.3390/molecules26185693)
Supplement: Supplementary file 1 [file molecules-26-05693-s001.zip › molecules-1306073-supplementary.pdf]

Supplementary Materials

Controlling the Substrate Specificity of an Enzyme through Structural Flexibility by Varying the Salt-Bridge Density

Juan Huang [1,2,†], Qin Xu [1,†,*], Zhuo Liu [2,3,4], Nitin Jain [5], Madhusudan Tyagi [6,7], Dong-Qing Wei [1,8,*], Liang Hong [2,3,*]

1 State Key Laboratory of Microbial Metabolism, School of Life Sciences and Biotechnology, Shanghai Jiao Tong University, Shanghai 200240, China; juanhuang2015@sjtu.edu.cn
2 Institute of Natural Sciences, Shanghai Jiao Tong University, Shanghai 200240, China
3 School of Physics and Astronomy, Shanghai Jiao Tong University, Shanghai 200240, China; liuzhuo-chirality@hotmail.com
4 Institute for Advanced Study, The Hong Kong University of Science and Technology, Hong Kong, China
5 Department of Biochemistry and Cellular and Molecular Biology, University of Tennessee, Knoxville, TN 37996, USA; njain@utk.edu
6 NIST Center for Neutron Research, National Institute of Standards and Technology (NIST), Gaithersburg, MD 20899, USA; madhusudan.tyagi@nist.gov
7 Department of Materials Science and Engineering, University of Maryland, College Park, MD 20742, USA
8 Peng Cheng Laboratory, Shenzhen 518055, China
* Correspondence: hongl3liang@sjtu.edu.cn (L.H.); dqwei@sjtu.edu.cn (D.-Q.W.); xuqin523@sjtu.edu.cn (Q.X.); Tel.: +86-213-420-4185 (Q.X.); +86-213-420-4573 (D.-Q.W.); +86-215-474-2996 (L.H.)
† These authors contributed equally to this work.

TABLE OF CONTENTS

1. Figure S1. S2
2. Figure S2. S3
3. Figure S3. S4
4. Figure S4 S5
5. Figure S5 S6
6. Table S1. S7
7. Table S2. S8
8. Table S3. S12
9. References S13

Protein	CYP101								CYP2C9							
PDB_ID	5GXG	2ZAX	1DZ9	5CP4	1PHC	2H7R	1P2Y	3L61	1R9O	5X23	5XXI	5W0C	5K7K	5A5J	5X24	5A5I
Substrate	DTT	CAM	CAM	CAM	0	1MZ	NCT	0	FLP	LSN	LSN	9W6	6RJ	6YF	LSN	XI1

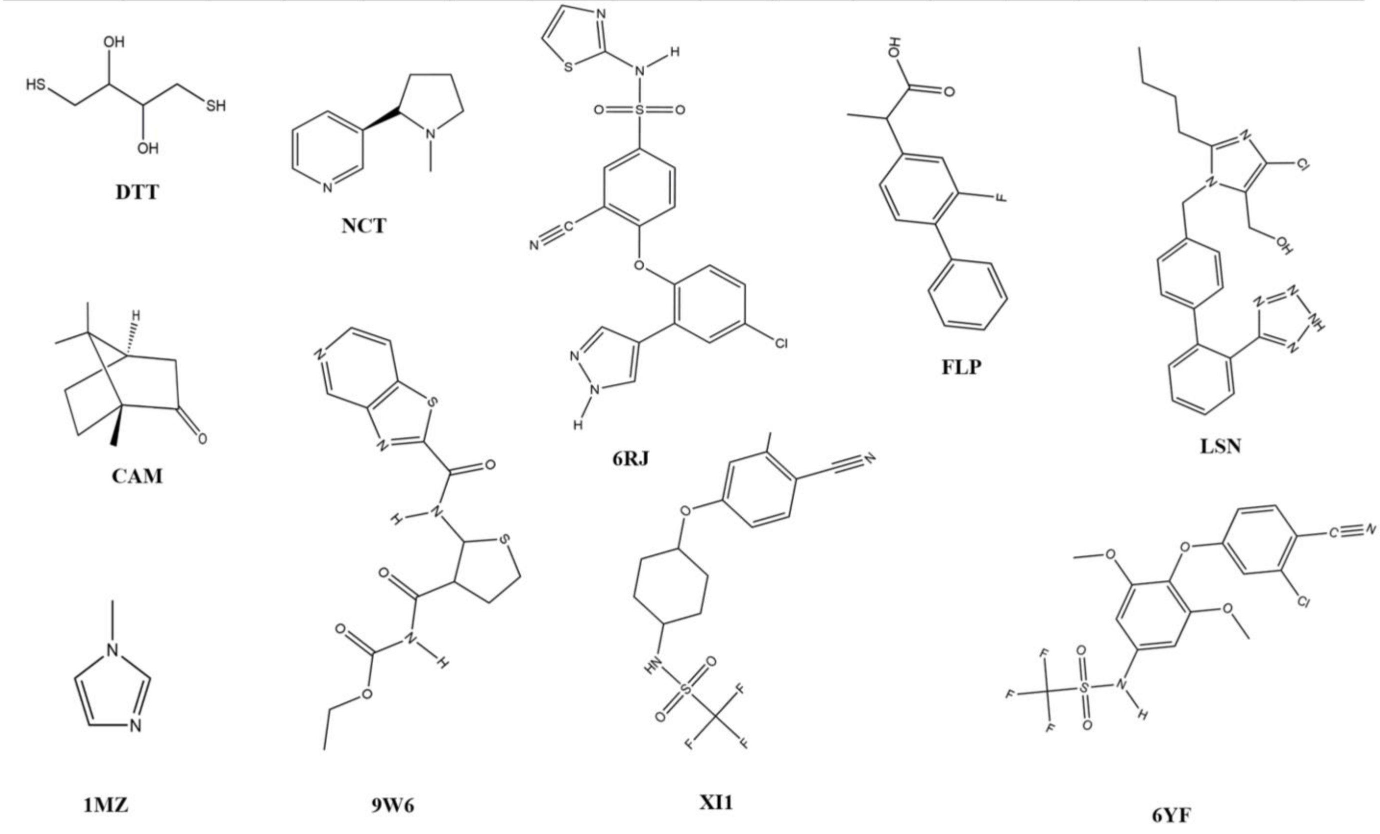

Figure S1. Detailed ligand information on selected crystal structures of CYP101 and CYP2C9 in Figure 2C, “0” represents a ligand-free structure.

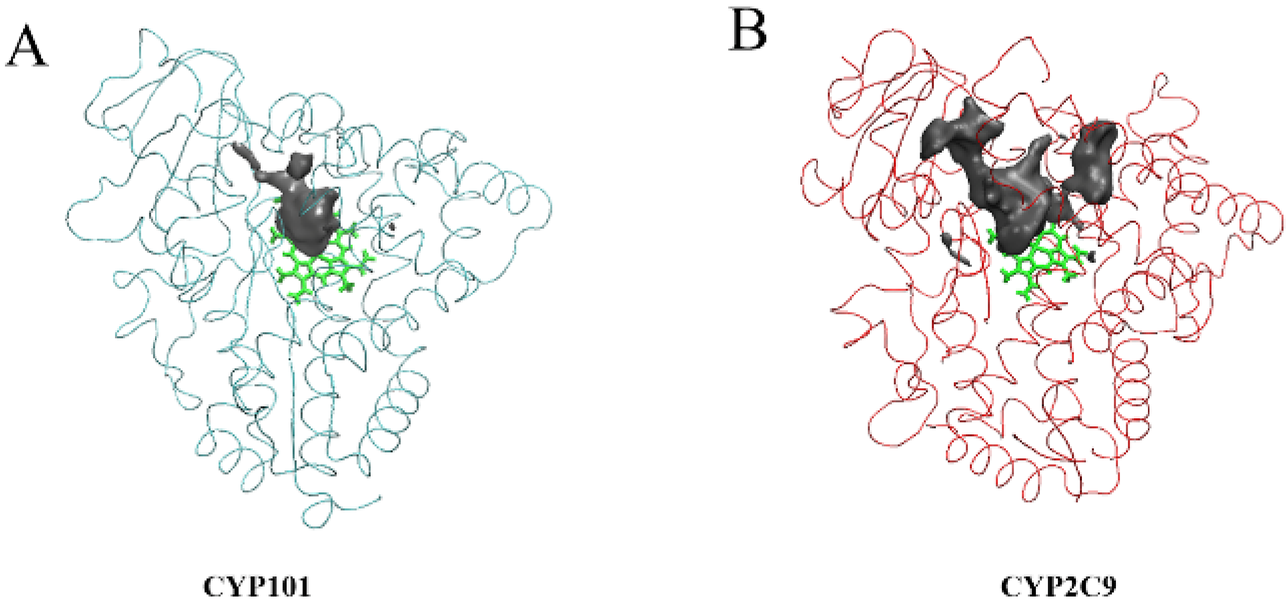

Figure S2. An example to compare the catalytic pockets between (A) CYP101 (PDB: 1DZ9) and (B) CYP2C9 (PDB: 5XXI). The pocket is calculated by the software POVME 2.0 [1, 2] and visualized by VMD [3].

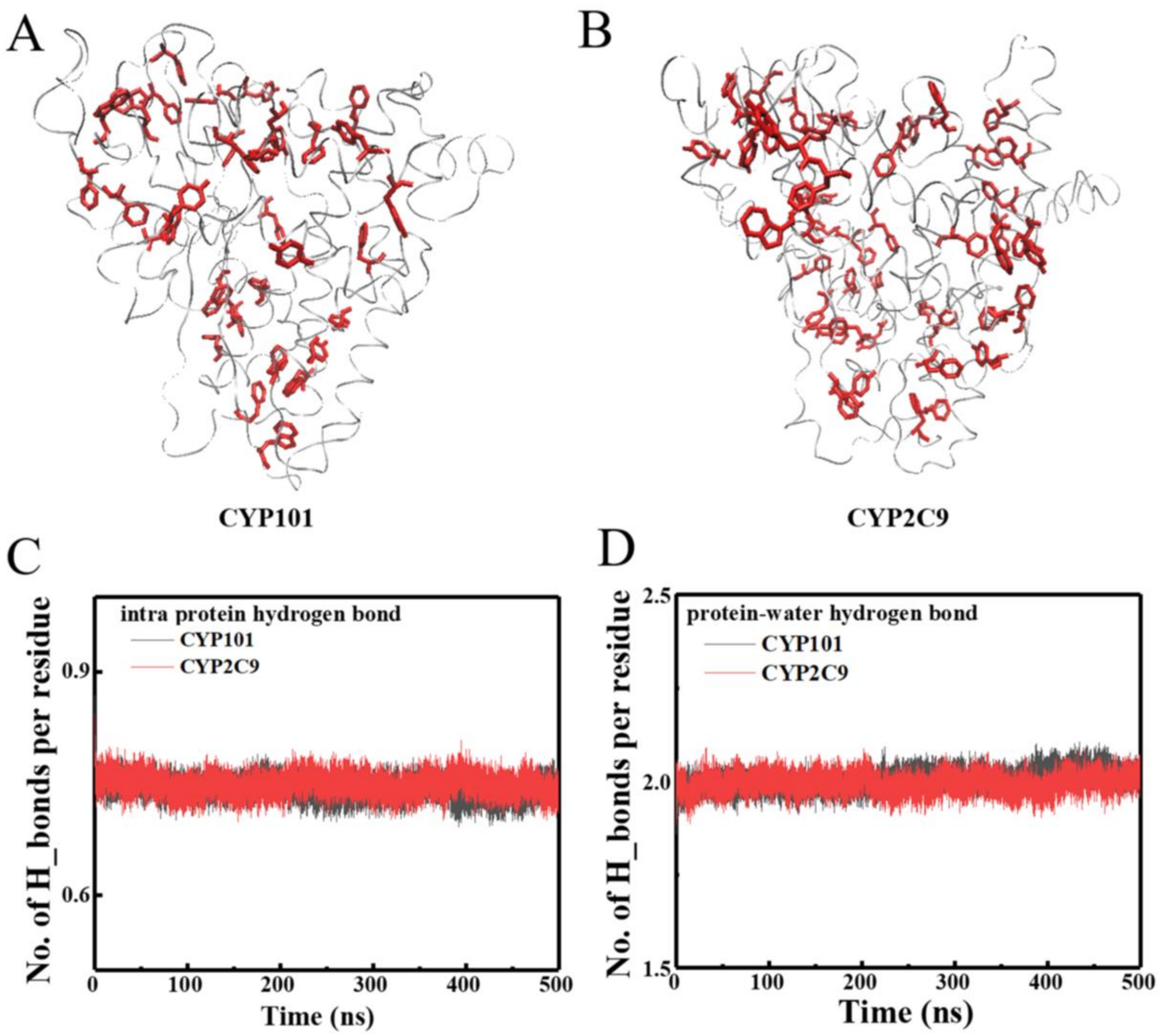

Figure S3. Comparison of other structural factors which might contribute to the difference in flexibility between CYP101 and CYP2C9. Distribution of aromatic residues (marked red) in (A) CYP101 (PDB: 1DZ9) and (B) CYP2C9 (PDB: 5XXI) in the two proteins (7.73% of CYP101 vs 9.59% of CYP2C9, and more details can be seen in Table S1). The hydrophobic interactions between nearby aromatic residues, or namely aromatic clusters, are often considered to play an important role in stabilizing and rigidifying the protein structure [4-6]. As seen in Figure S3A-B and Table S1, CYP2C9 has similar or slightly more aromatic residues as compared to CYP101. If the aromatic interaction plays a dominant role, CYP2C9 should have similar or even lower flexibility, contradicting our experimental and simulation observations (Figures 2 and 3 in the main text). The number of (C) intra-protein hydrogen bonds and (D) protein-water hydrogen bonds per protein residue for CYP101 (black) and CYP2C9 (red) are also quite similar for the two enzymes. Hence, all these interactions cannot be the dominant cause for the different structural flexibilities between the two CYP450s.

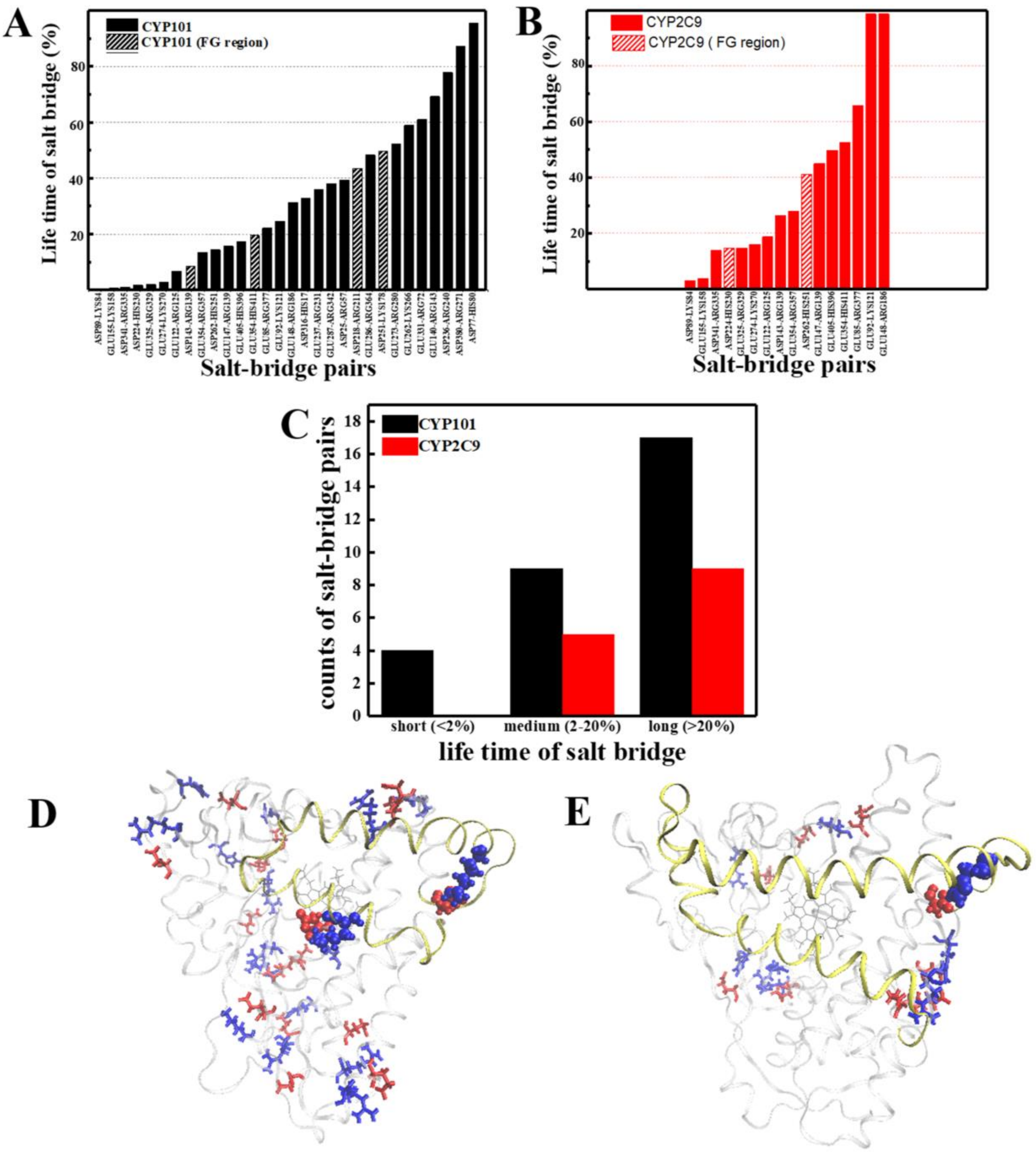

Figure S4. The life time of salt-bridge pairs in (A) CYP101 and (B) CYP2C9 obtained in 500 ns MD simulations. Those pairs at the F-G region are highlighted with slash. (C) The life time of salt-bridge pairs distribution in CYP101 and CYP2C9. The slat-bridge pairs at the F-G region and with life time higher than 20% (100 ns) are marked as bonds in the crystal protein structures of (D) CYP101 (PDB: 1DZ9) and (E) CYP2C9 (PDB: 5XXI), where the salt-bridge pairs in the F-G region are highlighted as spheres. The negatively and positively charged residues are colored in red and blue, respectively.

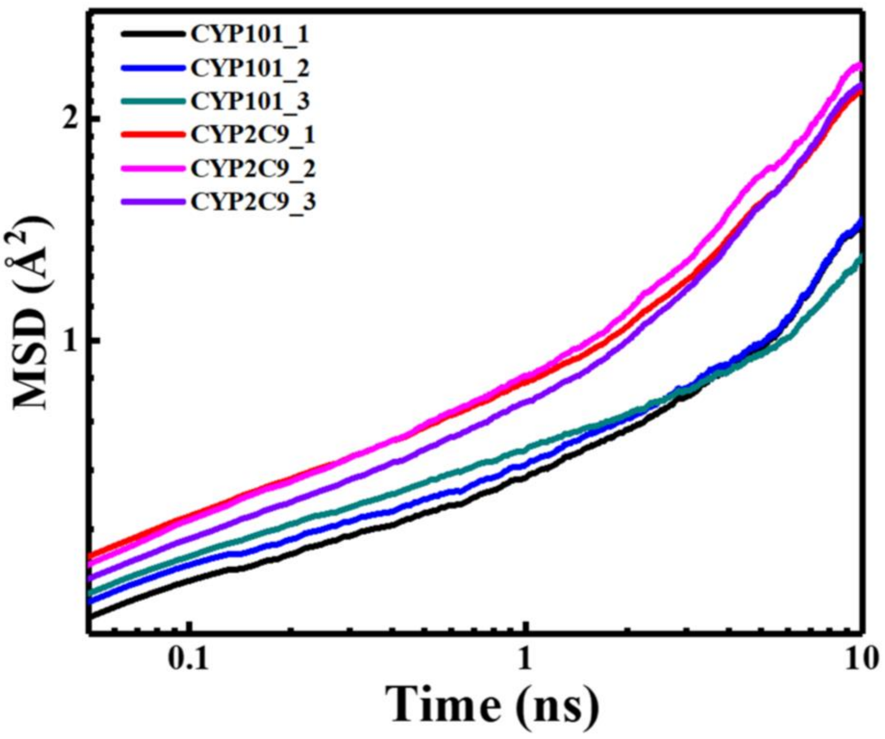

Figure S5. MD-derived mean-squared atomic displacement (MSD) (10 ps to 10 ns) in CYP101 and CYP2C9 obtained by analyzing three independent 500 ns simulations for each protein.

Table S1. Aromatic residues in CYP101 (PDB: 1DZ9) and CYP2C9 (PDB: 5XXI).

Protein	CYP101	CYP2C9
Trp	5	2
Tyr	9	12
Phe	18	33
Total aromatic residues	32	47
Total residues	414	490
Ratio	7.73%	9.59%

Table S2. List of 146 different kinds of CYP450s, whose experimentally determined structures were downloaded from the protein data bank and used for further MD simulations. The obtained MD results are presented in Figure 5.

Biological Categories	Organism(s)	CYP450s	PDB ID
Animalia	*bos taurus*	CYP11A1	3mzs
Animalia	*bos taurus*	CYP21A2	3qz1
Animalia	*danio rerio*	CYP17A1	4r1z
Animalia	*danio rerio*	CYP17A2	4r20
Animalia	*danio rerio*	CYP8A1	3b98
Animalia	*homo sapiens*	CYP11A1	3n9z
Animalia	*homo sapiens*	CYP11B1	6m7x
Animalia	*homo sapiens*	CYP11B2	4fdh
Animalia	*homo sapiens*	CYP17A1	5irv
Animalia	*homo sapiens*	CYP19A1	5jl7
Animalia	*homo sapiens*	CYP1A1	6dwn
Animalia	*homo sapiens*	CYP1A2	2hi4
Animalia	*homo sapiens*	CYP1B1	3pm0
Animalia	*homo sapiens*	CYP21A2	5vbu
Animalia	*homo sapiens*	CYP2A13	3t3s
Animalia	*homo sapiens*	CYP2A6	4rui
Animalia	*homo sapiens*	CYP2B6	4zv8
Animalia	*homo sapiens*	CYP2C19	4gqs
Animalia	*homo sapiens*	CYP2C8	2nni
Animalia	*homo sapiens*	CYP2C9	1og2
Animalia	*homo sapiens*	CYP2D6	4wnw
Animalia	*homo sapiens*	CYP2E1	3koh
Animalia	*homo sapiens*	CYP2R1	3czh
Animalia	*homo sapiens*	CYP3A4	4ny4
Animalia	*homo sapiens*	CYP3A5	6mjm
Animalia	*homo sapiens*	CYP46A1	2q9g
Animalia	*homo sapiens*	CYP51	3ld6
Animalia	*homo sapiens*	CYP7A1	3v8d
Animalia	*homo sapiens*	CYP8A1	2iag
Animalia	*neotoma lepida*	CYP2B37	5e0e
Animalia	*oryctolagus cuniculus*	CYP2B4	3tk3
Animalia	*oryctolagus cuniculus*	CYP2C5	1dt6
Animalia	*oryctolagus cuniculus*	CYP4B1	6c94
Animalia	*rattus norvegicus*	CYP24A1	3k9v
Archaeaa	*sulfurisphaera tokodaii*	CYP119	3b4x
Archaeaa	*picrophilus torridus*	CYP231A2	2rfb
Archaeaa	*sulfolobus acidocaldarius*	CYP119	1io8
Bacteria	*actinoplanes teichomyceticus*	CYP165D3	3o1a
Bacteria	*actinoplanes teichomyceticus*	CYPOxyA	5hh3
Bacteria	*actinoplanes teichomyceticus*	CYPOxyB	4tvf
Bacteria	*amycolatopsis balhimycina*	CYPOxyD	3mgx
Bacteria	*amycolatopsis mediterranei*	CYPRif16	5ysm
Bacteria	*amycolatopsis methanolica*	CYPGcoA	5omr

Bacteria	*amycolatopsis orientalis*	CYP105AS1	4oqs
Bacteria	*amycolatopsis orientalis*	CYPOxyB	1lgf
Bacteria	*amycolatopsis orientalis*	CYPOxyC	1ued
Bacteria	*arthrobacter sp*	CYP1232A24	6g71
Bacteria	*bacillus megaterium*	CYP106A2	5xnt
Bacteria	*bacillus megaterium*	CYP109A2	5ofq
Bacteria	*bacillus megaterium*	CYP109E1	5l90
Bacteria	*bacillus megaterium*	CYPBM3	3kx4
Bacteria	*bacillus methanolicus*	CYP152K6	6fyj
Bacteria	*bacillus subtilis*	CYP109B1	4rm4
Bacteria	*bacillus subtilis*	CYP134A1	3nc5
Bacteria	*bacillus subtilis*	CYP152A1	2zqj
Bacteria	*bacillus subtilis*	CYPBiol	3ejb
Bacteria	*bacillus subtilis*	CYPPksS	4yzr
Bacteria	*chondromyces apiculatus*	CYP109Q5	6gmf
Bacteria	*citrobacter braakii*	CYPcin	1t2b
Bacteria	*corynebacterium glutamicum*	CYPCREJ	5gwe
Bacteria	*exiguobacterium sp*	CYP152N1	5yhj
Bacteria	*jeotgalicoccus sp. 8456*	CYP152L1	4l54
Bacteria	*micromonospora griseorubida*	CYPMycCI	5foi
Bacteria	*micromonospora griseorubida*	CYPMycG	2y5n
Bacteria	*mycobacterium marinum*	CYP124A1	6cvc
Bacteria	*mycobacterium marinum*	CYP150A6	6dcd
Bacteria	*mycobacterium marinum*	CYP268A2	6bld
Bacteria	*mycobacterium smegmatis*	CYP125A3	5dqn
Bacteria	*mycobacterium smegmatis*	CYP142A	4uax
Bacteria	*mycobacterium smegmatis*	CYP164A2	3r9c
Bacteria	*mycobacterium tuberculosis*	CYP121	3g5f
Bacteria	*mycobacterium tuberculosis*	CYP124	2wm5
Bacteria	*mycobacterium tuberculosis*	CYP125	2x5l
Bacteria	*mycobacterium tuberculosis*	CYP126A1	5li7
Bacteria	*mycobacterium tuberculosis*	CYP130	2uuq
Bacteria	*mycobacterium tuberculosis*	CYP142	2xkr
Bacteria	*mycobacterium tuberculosis*	CYP144A1	5hdi
Bacteria	*mycobacterium tuberculosis*	CYP51	2bz9
Bacteria	*nocardia farcinica*	CYP154C5	4j6c
Bacteria	*nonomuraea recticatena*	CYP105	2z36
Bacteria	*novosphingobium aromaticivorans*	CYP101D1	4c9m
Bacteria	*novosphingobium aromaticivorans*	CYP101D2	3nv5
Bacteria	*novosphingobium aromaticivorans*	CYP108D1	3tkt
Bacteria	*pseudomonas putida*	CYP101A1	1dz9
Bacteria	*pseudonocardia autotrophica*	CYPvdh	5gnm
Bacteria	*rhodococcus erythropolis*	CYP1050A1	3wec
Bacteria	*rhodococcus rhodochrous*	CYPXplA	2wiv
Bacteria	*rhodopseudomonas palustris*	CYP199A2	2fr7
Bacteria	*novosphingobium aromaticivorans*	CYP101D2	3nv5
Bacteria	*saccharopolyspora erythraea*	CYPEryF	1z8p

Bacteria	*saccharopolyspora erythraea*	CYPEryK	2wio
Bacteria	*sorangium cellulosum*	CYP260A1	6f8a
Bacteria	*sorangium cellulosum*	CYP260B1	5hiw
Bacteria	*sorangium cellulosum*	CYP267B1	6gk5
Bacteria	*sorangium cellulosum*	CYPepok	1q5e
Bacteria	*sphingobium yanoikuyae*	CYP101J2	5kyo
Bacteria	*sphingomonas paucimobilis*	CYP152B1	3voo
Bacteria	*sphingomonas sp*	CYP153D17	5h1z
Bacteria	*sphingopyxis macrogoltabida*	CYPoyr	3rwl
Bacteria	*streptomyces acidiscabies*	CYPTxtC	6f0c
Bacteria	*streptomyces antibioticus*	CYPOleP	4xe3
Bacteria	*streptomyces arenae*	CYPPntM	5l1o
Bacteria	*streptomyces atroolivaceus*	CYPLnmA	4z5p
Bacteria	*streptomyces atroolivaceus*	CYPLnmZ	4z5q
Bacteria	*streptomyces avermitilis*	CYP105D6	3abb
Bacteria	*streptomyces avermitilis*	CYP105D7	4ubs
Bacteria	*streptomyces avermitilis*	CYP105P1	3e5j
Bacteria	*streptomyces avermitilis*	CYP107L2	5cje
Bacteria	*streptomyces avermitilis*	CYP107W1	4wpz
Bacteria	*streptomyces coelicolor*	CYP105N1	3tyw
Bacteria	*streptomyces coelicolor*	CYP154A1	1odo
Bacteria	*streptomyces coelicolor*	CYP154C1	1gwi
Bacteria	*streptomyces coelicolor*	CYP158A1	2nza
Bacteria	*streptomyces coelicolor*	CYP158A2	5de9
Bacteria	*streptomyces fradiae*	CYPTylHI	6b11
Bacteria	*streptomyces graminofaciens*	CYPGfsF	5y1i
Bacteria	*streptomyces griseolus*	CYP105A1	3cv8
Bacteria	*streptomyces griseoviridis*	CYPSGVP	4mm0
Bacteria	*streptomyces himastatinicus*	CYP107B1	4e2p
Bacteria	*streptomyces himastatinicus*	CYPHmtS	5z9i
Bacteria	*streptomyces himastatinicus*	CYPHmtT	4ggv
Bacteria	*streptomyces natalensis*	CYPPimD	2x9p
Bacteria	*streptomyces peucetius*	CYP105P2	5it1
Bacteria	*streptomyces scabiei*	CYPTxtE	4tpo
Bacteria	*streptomyces sp*	CYP163B3	4pxh
Bacteria	*phenylobacterium zucineum*	CYP153	6hqg
Bacteria	*Streptomyces sp*	CYP245A1	2z3t
Bacteria	*streptomyces sp. JS01*	CYP154C4	6a7i
Bacteria	*streptomyces thioluteus*	CYPAurH	3p3x
Bacteria	*streptomyces toyocaensis*	*CYPStaF*	*5ex9*
Bacteria	*streptomyces toyocaensis*	*CYPStaH*	*5ex6*
Bacteria	*streptomyces venezuelae*	*CYPPikC*	*2vzm*
Bacteria	*streptomyces violaceoruber*	*CYP154C4*	*6a7j*
Bacteria	*tepidiphilus thermophilus*	CYP116B46	6gii
Bacteria	*thermobispora bispora*	CYPTbtJ1	5vws
Bacteria	*Zobellia galactanivorans*	CYPZoGa	6g5q
Fungi	*candida albicans*	CYP51	5tz1

Fungi	*fusarium oxysporum*	CYP55A1	1cl6
Fungi	*candida glabrata*	CYP51	5jlc
Fungi	*neosartorya fumigata*	CYP51B	4uym
Fungi	*saccharomyces cerevisiae*	CYP51	5esn
Plantae	*parthenium argentatum*	CYP74A	3dam
Plantae	*salvia miltiorrhiza*	CYP76AH1	5ylw
Plantae	*arabidopsis thaliana*	CYP74A	3cli
Protista	*trypanosoma cruzi*	CYP51	5ajr
Protista	*trypanosoma brucei brucei*	CYP51	3g1q

Table S3. List of the Bacteria-like animal CYP450s in Figure 5

Organism[a]	CYP450	PDB ID	Salt bridge density	RMSF (nm)	Tissue	Sub-cellular[b]	Function	Chromosome
H	CYP19A1	5jl7	3.8	0.061	Widely	E	Estrogen biosynthesis	9
H	CYP7A1	3v8d	4.1	0.068	liver	E	Endogenous sterols	8
H	CYP11B2	4fdh	2.7	0.065	adrenal gland	M	Aldosterone-synthesizing	8
H	CYP51	3ld6	4.4	0.060	testis	E	Lanosterol 14-alpha demethylase	7
H	CYP11A1	3n9z	5.3	0.061	adrenal gland	M	Pregnenolone biosynthesis	15
H	CYP46A1	3mdt	4.9	0.063	brain	E	bile acids biosynthesis	14
H	CYP8A1	2iag	4.8	0.069	ovary	E	Prostacyclin synthase	20
H	CYP11B1	6m7x	3.36	0.064	adrenal gland	M	Glucocorticoid biosynthesis	8
D	CYP17A1	4r1z	3.3	0.071	adrenal gland	E	Androgen biosynthesis	13
R	CYP24A1	3k9v	4.4	0.068	kidney	M	vitamin D synthesis	3
B	CYP11A1	3mzs	2.9	0.060	adrenal gland	M	Cholesterol side-chain cleavage	8

[a]B: bos Taurus, D: danio rerio, H: homo sapiens, R: rattus norveqicus;

[b]E: endoplasmic reticulum, M: mitochondria;

REFERENCES

1. Durrant, J. D.; Votapka, L.; Sørensen, J.; Amaro, R. E., POVME 2.0: An Enhanced Tool for Determining Pocket Shape and Volume Characteristics. J. Chem. Theory Comput. 2014, 10, (11), 5047-5056.
2. Durrant, J. D.; de Oliveira, C. A. F.; McCammon, J. A., POVME: An algorithm for measuring binding-pocket volumes. Journal of Molecular Graphics and Modelling 2011, 29, (5), 773-776.
3. Humphrey, W.; Dalke, A.; Schulten, K., VMD: Visual molecular dynamics. Journal of Molecular Graphics 1996, 14, (1), 33-38.
4. Burley, S. K.; Petsko, G. A., Aromatic-aromatic interaction: a mechanism of protein structure stabilization. Science 1985, 229, (4708), 23.
5. Aravinda, S.; Shamala, N.; Das, C.; Sriranjini, A.; Karle, I. L.; Balaram, P., Aromatic−Aromatic Interactions in Crystal Structures of Helical Peptide Scaffolds Containing Projecting Phenylalanine Residues. J. Am. Chem. Soc. 2003, 125, (18), 5308-5315.
6. Liu, Z.; Lemmonds, S.; Huang, J.; Tyagi, M.; Hong, L.; Jain, N.; Entropic contribution to enhanced thermal stability in the thermostable P450 CYP119, Proc. Natl. Acad. Sci. U.S.A. 2018, 115 (43), E10049-E10058.
7. Karshikoff, A.; Jelezarov, I., Salt Bridges and Conformational Flexibility: Effect on Protein Stability. Biotechnol. Biotechnol. Equip. 2008, 22, (1), 606-611.
